# Human T-Lymphotropic Virus Type 1–Associated Myelopathy With Autoimmune Cholangiopathy: An Unusual Immune Conundrum in a Young Patient

**DOI:** 10.1155/crdi/7381720

**Published:** 2025-09-08

**Authors:** Aida Saad, Yamna Jadoon, Riffat Sabir

**Affiliations:** ^1^Department of Medicine, University of Massachusetts Chan Medical School-Baystate Regional Campus, Springfield, Massachusetts, USA; ^2^Department of Hematology & Oncology, University of California, San Francisco, California, USA

**Keywords:** autoimmune cholangiopathy, autoimmune hepatitis-primary sclerosing cholangitis overlap syndrome, fever of unknown origin, HTLV-1, HTLV-1 myelopathy

## Abstract

Human T-lymphotropic virus type 1 (HTLV-1), the first oncogenic retrovirus discovered in humans, is primarily associated with two disease entities: adult T cell leukemia-lymphoma and HTLV-1-associated myelopathy-tropical spastic paresis. HTLV-1 has also been implicated in the pathogenesis of various autoimmune rheumatic diseases, and its association with the autoimmune disorders of the gastrointestinal track is less well understood. Our patient, a 26-year-old previously healthy female, presented with recurrent, progressively worsening chronic abdominal pain and persistent liver test abnormalities. Initially diagnosed with acute acalculous cholecystitis and autoimmune hepatitis (AIH), her liver tests continued to exhibit a predominantly cholestatic pattern. This prompted further advanced imaging, and magnetic resonance cholangiopancreatography ultimately confirmed a diagnosis of primary sclerosing cholangitis (PSC). Complicating her condition further, she developed lower extremity weakness, initially attributed to axonal Guillain–Barré syndrome, which unfortunately did not respond to standard treatment. After a year marked by progressive clinical decline with repeated and prolonged hospitalizations due to fever of unknown origin, an extensive diagnostic workup ultimately led to a diagnosis of HTLV-1 myelopathy, along with AIH-PSC overlap syndrome. This case highlights the diagnostic challenges associated with the multisystem involvement of HTLV-1. Notably, our patient's presentation was not consistent with classic HTLV-1 myelopathy rather a subtype with rapidly progressive symptoms and flaccid as opposed to spastic paresis. The association between HTLV-1 infection and autoimmune cholangiopathy is exceptionally rare. To the best of our knowledge, our case represents only the second reported instance of autoimmune cholangiopathy associated with HTLV-1 myelopathy and the first reported case of AIH-PSC overlap syndrome associated with HTLV-1 myelopathy. This underscores the need for heightened clinical awareness of potential hepatic immune manifestations in patients with HTLV-1 infection, even in the absence of classic neurologic symptoms at initial presentation.

## 1. Introduction

Despite being one of the first discovered oncogenic human retroviruses, human T-lymphotropic virus type 1 (HTLV-1) remains an elusive entity [1]. Endemic to Central and West Africa, the Middle East, Melanesia, Australia, Japan, the Caribbean, and South America, this virus is present throughout the world with an underrecognized disease burden. It has been estimated to infect 5–10 million people worldwide, mode of transmission being sexual, transplacental, breastfeeding, and blood transfusions—particularly in patients who received transfusions prior to the 1980s [2, 3].

While most individuals infected with HTLV-1 remain asymptomatic, a subset develop serious complications, including adult T-cell leukemia-lymphoma (ATLL) and HTLV-1-associated myelopathy-tropical spastic paraparesis (HAM-TSP) [4]. HTLV-1 has also been implicated in the pathogenesis of many autoimmune diseases, i.e., diabetes, multiple sclerosis, infective dermatitis, and uveitis as well as rheumatic diseases including rheumatoid arthritis, Sjögren's syndrome, systemic lupus erythematosus, and polymyositis [5]. However, its association with autoimmune disorders of hepatic and biliary track is less well understood.

We present a 26-year-old female who developed HAM-TSP, alongside an autoimmune hepatitis-primary sclerosing cholangitis (AIH-PSC) overlap syndrome, which took over a year to accurately diagnose. In this case report, we aim to explore potential link between HTLV-1 infection and autoimmune cholangiopathy, shedding light on possible complex connections between chronic viral infections and autoimmune processes. This case also underscores the importance of recognizing HTLV-1 as a potential contributor in autoimmune hepatic and biliary pathologies, expanding the spectrum of its clinical manifestations.

## 2. Case Presentation

A 26-year-old female with no known medical comorbidities presented to our emergency department (ED) with a 10-day history of vomiting and epigastric abdominal pain. Laboratory workup revealed elevated liver function tests (LFTs) (alkaline phosphatase [ALP] 908 U/L, aspartate aminotransferase [AST] 97 U/L U/L, alanine transaminase [ALT] 144 U/L, and total bilirubin 1.3 mg/dL), mildly elevated white blood cell (WBC) count of 12.9 k/μL, and a negative serum pregnancy testing. A week prior, she had presented to an outside hospital (OSH) with similar complaints. Computed tomography (CT) scan of the abdomen and pelvis (CT A/P) with intravenous (IV) contrast revealed extensive mesenteric lymphadenopathy, prompting a lymph node biopsy that showed reactive tissue. The transaminitis was attributed to her prescribed antifungal medication for a superficial toe fungal infection and nonspecific viral infection. A repeat CT A/P at our facility confirmed these findings, and subsequent LFTs showed a downtrending pattern, correlating with her symptomatic improvement after IV hydration and analgesics (tramadol 50 mg as needed × 5 days), after which she was discharged home.

One month later, the patient was readmitted to an OSH with abdominal pain and was diagnosed with acute acalculous cholecystitis. She was treated with IV antibiotics (piperacillin/tazobactam 3.337 mg Q6H × 5 days) and underwent a cholecystectomy and liver biopsy. Pathology of the gallbladder showed chronic cholecystitis. Pathology of liver biopsy showed moderate chronic hepatitis with dense portal lymphoplasmacytic infiltrate with moderate interface activity, moderate lobular inflammation and ballooned hepatocytes with portal and periportal fibrosis, and minimal intrahepatic cholestasis. During this hospitalization, she also underwent a substantial workup, which was negative and included testing for hepatitis A, hepatitis B, hepatitis C, human immunodeficiency virus (HIV), cytomegalovirus (CMV), antinuclear antibodies (ANAs), and anti-mitochondrial antibodies (AMAs). AIH was suspected, for which she was started on steroids (prednisone 40 mg daily, with plans for tapering in the outpatient setting) and subsequently discharged home after improvement of symptoms. Soon after discharge, the patient developed gradually worsening lower extremity weakness, eventually resulting in an inability to ambulate, necessitating a readmission 2 weeks later. The details of this hospitalization at the OSH are unclear, but allegedly, the patient received antibiotics for a suspected infection. Her persistent weakness was attributed to deconditioning, prednisone 40 mg daily was continued, and she was transferred to a rehabilitation facility.

At the facility, the patient's lower extremity weakness persisted without improvement. However, after experiencing generalized tonic-clonic seizures, she represented to our hospital for further evaluation. Her neurological examination was consistent with a bilateral, flaccid paralysis with a sensory level between the umbilicus and T4. Upper extremity motor power was rated 4/5 compared to 1–2/5 in the lower extremities, accompanied by bilateral absence of patellar reflexes. Laboratory workup showed a WBC count of 42 k/μL with elevated absolute neutrophilia and lymphocytosis, mild normocytic anemia with a hemoglobin level of 11.5 g/dL, and thrombocytosis with a platelet count of 931 k/mm^3^. Her LFTs were mildly elevated but stable (ALP 767, AST 92, ALT 112 U/L, and total bilirubin 1.3 mg/dL) compared to previous lab work.

**Figure 1:**
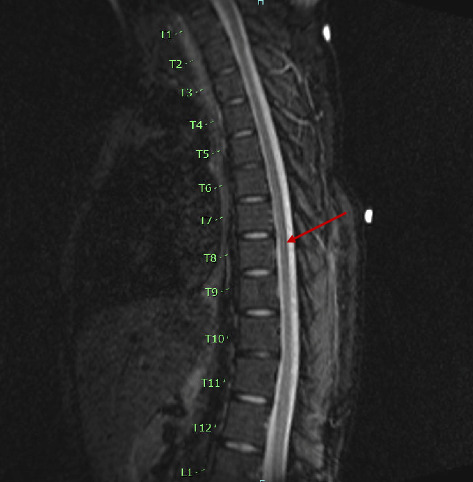
Magnetic resonance imaging of the thoracic spine demonstrating diffuse contrast enhancement of cauda equina nerve roots.

CT imaging of the head revealed no evidence of acute intracranial abnormality. However, the magnetic resonance imaging (MRI) of the cervical, thoracic, and lumbar spines was significant for diffuse cauda equina nerve root enhancement without any evidence of cord compression or spinal infection (Figure 1).

CSF analysis showed a mildly elevated total protein of 106 mg/dL with normal cellular counts, glucose levels, and a negative meningitis/encephalitis pathogen panel. Electromyography (EMG) nerve conduction study showed severe acute motor and sensory axonal polyneuropathy affecting the lower extremities more than the upper extremities, compatible with axonal Guillain–Barré syndrome (GBS) and acute motor and sensory axonal neuropathy. The patient received intravenous immunoglobulin (IVIg) therapy at 0.4 gm/kg per day for 5 days without any clinical improvement. A comprehensive diagnostic evaluation for heavy metal poisoning, myasthenia gravis, viral hepatitis, sarcoidosis, paraneoplastic autoantibody evaluation, and a hematological workup including a bone marrow biopsy to elucidate the cause of her complex clinical presentation and multiple laboratory derangements remained inconclusive (Table 1).

On day 17 of her hospitalization, she developed a 101.5°F fever, which continued to recur intermittently during the next several weeks. Blood and urine cultures returned negative. CT chest, abdomen, and pelvis revisualized previously observed mesenteric lymphadenopathy. A tagged WBC scan showed no abnormal uptake to suggest an occult infection. After she continued to remain febrile despite all measures, bromocriptine was initiated due to suspicion of central fever secondary to autonomic dysfunction. The patient's persistent leukocytosis and thrombocytosis (Table 2) were attributed to her chronic illness and ongoing steroid therapy, especially in the context of a negative extensive diagnostic workup that ruled out other potential causes.

**Figure 2:**
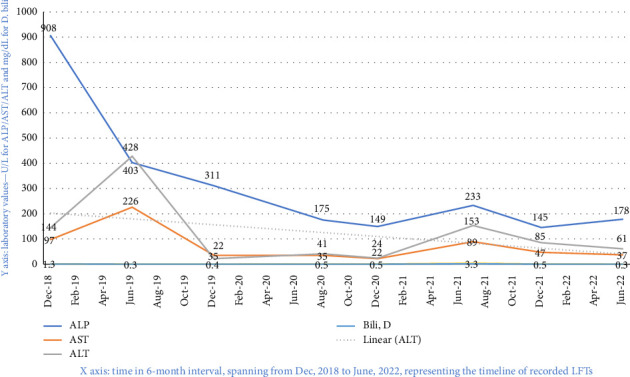
Liver function trends measured at 6-month intervals from years 2018–2022.

During this prolonged hospitalization, the patient was seen by gastroenterology for evaluation of long-standing transaminitis (Figure 2). Further testing, including repeat AMA, hepatitis B serum antigen, hepatitis B serum antibody, hepatitis C antibody, HIV Ab-Ag, and CMV testing, all returned negative. However, ANA screen returned positive with a titer of 1:80 and a homogenous pattern, along with an elevated total IgG count of 3600 mg/dL (normal range: 767–1590 mg/dL) and an elevated smooth muscle antibody (ASMA) level at 35U, but negative liver/kidney microsomal antibody. At this point, the leading differentials for the patient's transaminitis included autoimmune cholangiopathy and AIH; therefore, a tapering dose of steroids was continued. Her neurological function had been stable for approximately 1 month, with 3/5 strength in the upper extremities and 0–1/5 in the lower extremities, and a bedbound functional status. After a prolonged hospital stay spanning 73 days, she was discharged to a rehabilitation facility.

**Figure 3:**
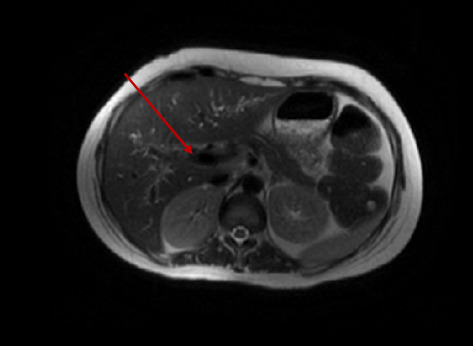
MRCP showing diffuse intrahepatic bile duct irregularities, multiple narrowings, and segmental dilations in the biliary tree, consistent with large duct primary sclerosing cholangitis.

A few weeks later, magnetic resonance cholangiopancreatography (MRCP) imaging was performed on an outpatient basis to evaluate for any biliary abnormalities. It revealed diffuse intrahepatic and extrahepatic biliary ductal dilatation with alternating areas of stricture and dilatation, consistent with large duct PSC (Figure 3). She subsequently underwent endoscopic retrograde cholangiopancreatography (ERCP), which revealed multiple severe diffuse biliary strictures and a single dominant stricture in the middle third of the common bile duct, for which sphincterotomy and biliary stenting were performed, resulting in resolution of hyperbilirubinemia. She was started on ursodiol (600 mg Q12H) and transferred to the liver transplant facility. Over the ensuing weeks, she underwent multiple ERCPs with biliary stent removals and replacements, conducted at both our facility and the transplant center; the family, however, declined evaluation for liver transplant, citing travel distance as a limiting factor.

The patient's subsequent hospitalizations unfolded in a series of complex events marked by recurrent symptoms and diagnostic challenges. One month later, she returned with abdominal pain and recurrent fevers; diagnostic workup was expanded to include viral, chronic bacterial, fungal, and parasitic infections (Table 3).

**Figure 4:**
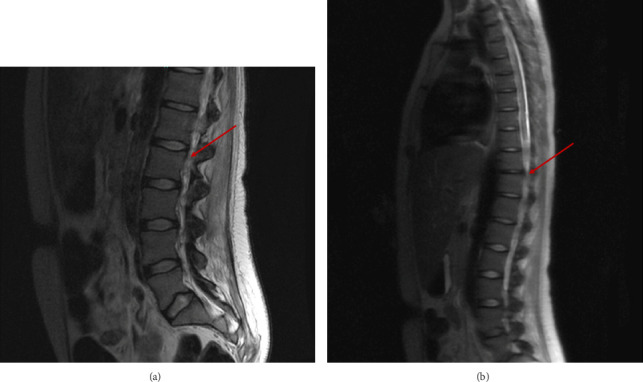
(a) Repeat MRI of the lumbar spine demonstrating diffuse spinal cord swelling with central T2 bright signal and peripheral enhancement. (b) Repeat MRI of the thoracic-lumbar spine demonstrating diffuse spinal cord swelling with central T2 bright signal and mild diffuse atrophy of the thoracic spinal cord—particularly in the mid-to-lower thoracic segments.

Workup returned positive for anti-HTLV-1 antibodies by western blot. Repeat MRI of the brain and cervical, thoracic, and lumbar spines showed diffuse patchy T2 hyperintensities throughout the white matter, in addition to spinal cord swelling in the cervical spine, suspected to be secondary to HTLV-1 myelopathy (Figures 4(a) and 4(b)). Lumbar puncture showed elevated CSF white cells with lymphocytic predominance (144/mm^3^, 96%), elevated total protein (121 mg/dL), and low glucose (31 mg/dL), and positive HTLV-1 antibody on western blot. Repeat EMG showed severe axonal polyneuropathy with diffuse active nerve degeneration. As classic HAM causes spastic paraparesis without sensory deficits, the patient's presentation with flaccid paraparesis and sensory deficits was atypical for the classic form of HAM.

**Figure 5:**
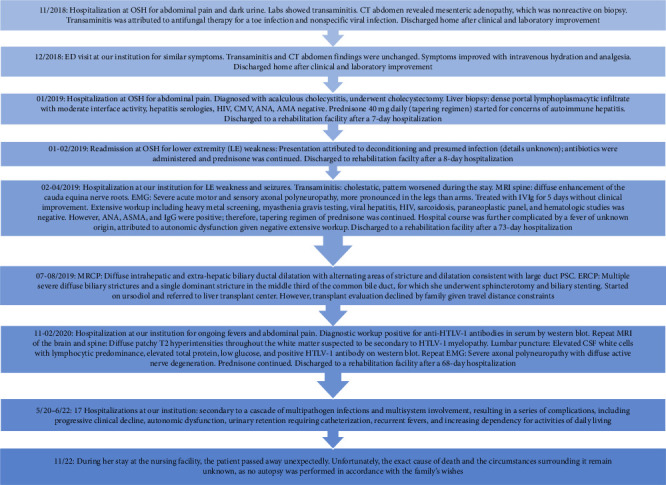
The timeline outlining patient's complex clinical course and multiple hospitalizations.

Over the next 2 years, the patient had recurrent hospitalizations as frequent as 1 or 2 per month. These hospitalizations were characterized by a cascade of multipathogen infections and multisystem involvement, resulting in a series of complications, including progressive clinical decline, autonomic dysfunction, urinary retention requiring catheterization, recurrent fevers, and increasing dependency for activities of daily living. The timeline outlining her complex clinical presentation is summarized in Figure 5.

While at the nursing facility, the patient passed away unexpectedly. Unfortunately, the exact cause of death and the circumstances surrounding it remain unknown, as no autopsy was performed in accordance with the family's wishes.

## 3. Discussion

This case highlights an intriguing yet diagnostically challenging case of a young female who was initially diagnosed with autoimmune cholangiopathy and later found to have HAM. The patient first presented with recurrent, progressively worsening chronic abdominal pain alongside persistent cholestatic liver enzyme abnormalities. Serologic workup revealed a positive ANA at 1:80 with a homogeneous pattern, elevated ASMA at 35 U, and a markedly elevated serum IgG concentration (3600 mg/dL). A liver biopsy demonstrated interface hepatitis and lobular inflammation, confirming the autoimmune nature of liver injury. MRCP revealed diffuse intrahepatic and extrahepatic bile duct dilation with alternating areas of stricture and dilatation, findings characteristic of large duct PSC. Together, the serologic, histologic, and radiologic features were deemed consistent with the diagnosis of AIH-PSC overlap syndrome, a rare entity in young patients. Concurrently, the patient developed progressive lower extremity weakness, urinary dysfunction, and fever of unknown origin. Spinal cord MRI revealed diffuse patchy T2 hyperintensities throughout the white matter, and serologic testing confirmed HTLV-1 infection, leading to a diagnosis of HTLV-1 myelopathy. Although HAM-TSP typically presents with spastic paraparesis without sensory deficits, the patient's presentation with flaccid paraparesis and sensory deficits was atypical for the classic form of HAM.

This case highlights the diagnostic challenges associated with the multisystem involvement of HTLV-1. The co-occurrence of HAM and AIH-PSC overlap syndrome raises important questions about the virus's broader immunopathologic impact. While HTLV-1 has been implicated in various autoimmune conditions, its association with autoimmune cholangiopathy is exceptionally rare. To the best of our knowledge, our case represents only the second reported instance of autoimmune cholangiopathy associated with HTLV-1 myelopathy and the first reported case of AIH-PSC overlap syndrome associated with HTLV-1 myelopathy, underscoring the need for heightened clinical awareness of potential hepatic and biliary immune manifestations in patients with HTLV-1 infection.

HAM-TSP is a rare chronic neurological disease caused by HTLV-1 characterized by marked progressive disability and poor quality of life [6]. Symptoms include spasticity, bladder or bowel sphincter dysfunction, paraparesis, hyperreflexia, extensor plantar reflex, ankle clonus, and back pain. Diagnosis involves clinical, laboratory, and imaging evaluation to confirm the presence of HTLV-1 infection. Detection of HTLV-1 antibodies in the CSF is indicative of central nervous system involvement; CSF may also reveal HTLV-1-specific oligoclonal bands and lymphocytic pleocytosis with an increase in CSF protein. MRI of the brain/spinal cord can reveal white matter lesions predominantly in the subcortical or periventricular areas as well as atrophy of the cervicothoracic cord [6]. During the diagnostic process, other causes of paraparesis should be thoroughly ruled out.

HTLV-1-infected patients may exhibit a wide variety of neurological manifestations distinct from the classical picture of HAM-TSP [7], making a diagnosis of this disease much more difficult. Due to the insidious nature of the disease and rarity, patients often undergo extensive workup before receiving a diagnosis [8]. Management of HTLV-1-related myelopathy remains challenging and without any curative agents. There have been success stories associated with the use of high-dose steroids and cyclophosphamide, with reported improvement in clinical symptoms within 3.5 years [9]. Steroids used as induction and maintenance therapies in patients with HAM-TSP are noted to improve gait function and slow the rate of disease progression [10, 11]. When steroids are contraindicated, alternative agents including antiretroviral therapies, interferon-gamma, and anti-CCR monoclonal antibodies can be used; however, the evidence supporting their effectiveness is limited [10].

HTLV-1 has also been implicated in the pathogenesis of various autoimmune diseases, i.e., diabetes, multiple sclerosis, infective dermatitis, uveitis, and arthropathies [5], hypothesized to arise from immune response dysregulation and molecular mimicry [12]. HTLV-1 infection has been previously linked with primary biliary cirrhosis (PBC) [12, 13], acute liver injury [14], and PSC [15]; however, but its connection to the AIH-PSC overlap syndrome remains speculative and poorly defined. The first case of a patient with HAM-TSP and PSC was reported in 1991 by Takegoshi et al. Similar to our patient, this individual was initially diagnosed with PSC and later developed a slowly progressive, symmetrical myelopathy, which ultimately led to an HTLV-1 diagnosis through serologic testing and imaging more than 2.5 years later [15]. This case suggested a potential association between HTLV-1 infection and PSC, also indicating a role of HTLV-1 in triggering the immune system's response in individuals genetically predisposed to PSC. Additionally, in another case by Mizuki et al, an HTLV-1 carrier was found to have PBC along with mixed connective tissue disease, also attributed to HTLV-1-related immune dysfunction [16]. A recent case report described acute liver failure in a patient diagnosed with ATLL and HTLV-1 infection. In this particular patient, ATLL arising from HTLV-1 infection was reported to infiltrate the liver, resulting in massive hepatic ischemia and widespread necrosis of the hepatocytes, leading to acute hepatitis and liver injury [17]. Given the paucity of reported cases, it is hard to determine whether the association between HTLV-1 infection and autoimmune cholangiopathy represents a true pathological link or a coincidental association. Previously published studies have speculated that an underlying immune dysregulation may play a role in such associations. However, further research studies are needed to clearly delineate potential immunologic or virologic mechanisms that may underlie this rare clinical constellation.

The diagnosis of the AIH-PSC overlap syndrome before HTLV-1 infection in our patient further adds weight to the hypothesis that HTLV-1 infection may act as a silent immunologic trigger, unmasking autoimmune disease in genetically predisposed individuals [18]. AIH-PSC overlap syndrome diagnosis requires integrating laboratory (elevated AST/ALT, γ-globulin, IgG, and GGT), serologic (negative AMA), histopathologic (interface hepatitis), and cholangiographic data (biliary strictures with intervening dilations), often in the context of a compatible clinical presentation [19]. Management of AIH-PSC overlap syndrome is derived from clinical anecdotes, observations, or retrospective case series. In PSC, management of dominant strictures typically involves endoscopic dilation with or without stenting. The role of ursodeoxycholic acid is controversial, but it may be considered at a low dose of 13–15 mg/kg/day in patients with significant cholestasis. Treatment of AIH involves immunosuppression, including corticosteroids, with azathioprine and mycophenolate mofetil used as steroid-sparing agents [19]. However, immunosuppression must be approached with caution in patients with HTLV-1 infection given concerns around viral reactivation and secondary infections. A multidisciplinary approach is essential to balance immune modulation with infection control and neurologic progression in a multisystem-involved HTLV-1.

## 4. Conclusion

Our case highlights the diagnostic challenges associated with the multisystem involvement of HTLV-1 infection, underscoring the importance of early detection of life-limiting diseases, which can significantly deteriorate one's quality of life and impact one's loved ones. Clinicians should maintain a high index of suspicion for HTLV-1 infection in patients with atypical autoimmune presentations, particularly when systemic symptoms and neurologic signs coexist, as early recognition would allow a palliative and symptom-centered approach that can improve patients' overall well-being.

The existing body of literature lacks a clear understanding of the potential association between HTLV-1 infection and autoimmune cholangiopathy. There is currently no established causal link, and reported cases, if any, may reflect unrecognized mechanisms of immune dysregulation. To elucidate this association, future studies could consider comparative analyses of liver biopsy samples and MRCP imaging from HTLV-1-infected patients with and without autoimmune cholangiopathy, which may help identify distinguishing features. Another future research direction includes identification of unique biomarkers facilitating earlier diagnosis and risk stratification of this potentially progressive disease. Lastly, exploring the molecular mechanisms linking HTLV-1 to autoimmune cholangiopathy through whole genome sequencing to uncover genetic or immunologic pathways that predispose individuals to this overlap syndrome can be another valuable direction. Although corticosteroids may slow disease progression and early data on the use of biologics in HAM-TSP are promising, further research is imperative to establish standard-of-care therapies and ultimately find a cure.

## Figures and Tables

**Table 1:** Comprehensive diagnostic workup conducted during hospitalization.

Laboratory test	Results	Reference value
*Heavy metal analysis*		
Lead/creatinine ratio	< 1	< 2 mcg/g Cr
Arsenic/creatinine ratio	< 2	< 24 mcg/g Cr
Cadmium/creatinine ratio	< 0.5	< 0.6 mcg/g Cr
Mercury/creatinine ratio	< 2	< 2 mcg/g Cr
Copper level	0.93	0.75–1.45 mcg/mL
Zinc level	1.26	0.66–1.10 mcg/mL

*Immunology testing*		
ACHR binding Ab	0	< EQ 0.02 nmol/L
Antinuclear Ab	Positive, homogenous 1:80	< 1:80
Liver/kidney microsomal Ab	< 5	< EQ 20 U
Mitochondrial Ab	< 20	0–20 U
Rheumatoid factor	< 10	< 14 IU/mL
Smooth muscle Ab	35	0–19 U
Total IgG	3600	767–1590 mg/dL
IgG subclass 1	2290	341–894 mg/dL
IgG subclass 2	1160	171–632 mg/dL
IgG subclass 3	210	18.4–106 mg/dL
IgG subclass 4	98.1	2.4–121 mg/dL

*Paraneoplastic autoantibody evaluation*		
Antineuronal nuclear antibody (ANNA)-1	Negative	< 1:240 titer
ANNA-2	Negative	< 1:240 titer
ANNA-3	Negative	< 1:240 titer
Anti-glial nuclear antibody (AGNA)-1	Negative	< 1:240 titer
Purkinje cell cytoplasmic antibody (PCA)-1	Negative	< 1:240 titer
PCA-2	Negative	< 1:240 titer
PCA-Tr	Negative	< 1:240 titer
Amphiphysin	Negative	< 1:240 titer
Collapsin response mediated protein (CRMP)-5	Negative	< 1:240 titer
Voltage gated calcium channel (VGCC) P/Q	0	< EQ 0.02
VGCC N	0.01	< EQ 0.03
AChR, ganglionic	0.01	< EQ 0.02
Voltage gated potassium channel (VGKC)	0.01	< EQ 0.02
Myelin associated Ab, IgM	Negative	Negative
Ganglioside (GM1), IgM and IgG	< 1:250	< EQ 1:1000
Asialo GM1 IgM and IgG	< 1:250	< EQ 1:4000
GD 1B, IgM and IgG	< 1:250	< EQ 1:1000
Striational Ab	1:120	< 1:120

*Viral serologies*		
Anti-hepatitis A IgM	Negative	Negative
Hepatitis B surface antigen	Negative	Negative
Hepatitis B core Ab, IgM	Negative	Negative
Anti-HBS quant	0.4	< 8.00 mIU/mL, indicates lack of prior exposure
Hepatitis E IgM	Negative	Negative
Hepatitis C Ab	Negative	Negative
Herpes simplex virus IgM	< 0.90	< 0.90
CMV IgG Ab	Negative	
HIV 4^th^ generation Ab-Ag	Negative	Negative

*Hematological workup*		
Flow cytometry	No monotypic B-cell population and no aberrant T-cell population identified	N/A
Myeloproliferative leukemia exon 10 mutational analysis	Negative	N/A
JAK2 V617F mutation analysis	Negative	N/A
Calreticulin (CALR) mutational analysis	Negative	N/A
BCR/ABL 1	Negative	N/A
Cytogenetics, chromosome analysis	Normal	N/A
Bone marrow biopsy	Normocellular marrow with normal hematopoiesis	N/A

**Table 2:** Serial laboratory test results over hospitalization.

Laboratory test	Day 1	Day 17	Day 45	Day 73 (discharge day)
Hemoglobin (gm/dL)	11.5	9.6	10.3	9.9
Hematocrit (%)	37.3	31.2	31.8	32.4
WBC (k/mm^3^)	42	17.3	15.7	20.6
Platelet count (k/mm^3^)	931	478	661	673
Alk phos (U/L)	766	330	1416	817
AST (U/L)	92	48	297	173
ALT (U/L)	112	66	458	305
T. bilirubin (mg/dL)	1.3	0.6	0.5	0.3
D. bilirubin (mg/dL)	0.5	0.2	0.1	0.1

**Table 3:** Comprehensive diagnostic workup conducted during hospitalization.

Laboratory test	Results	Reference value
*Autoantibody evaluation*		
PTT-LA (lupus sensitive)	64.8	0.0–59.1 s
Lupus anticoagulant	Not detected	N/A
LA-PTT-LA mix	57.2	0.0–48.9 s
Albumin SPE	2.8	3.1–4.8 Gm/dL
Alpha 1 SPE	0.3	0.2–0.4 Gm/dL
Alpha 2 SPE	0.8	0.6–0.9 Gm/dL
Beta SPE 1	1	0.8–1/3 Gm/dL
Gamma SPE	2.1	0.6–1.8 Gm/dL
C3 complement	99	90–180 mg/dL
C4 complement	29	10–40 mg/dL
Immunoglobulin IgE	9	0–199 IU/mL
Anti-native DNA	< 1	0–9
Rheumatoid factor	< 10	Normal < 14 IU/mL
Anti-SSA (Ro)	< 0.2	0.0–0.9 AI
Anti SSB (La) Ab	< 0.2	0.0–0.9 AI
Histone Ab	1.7	0.0–0.9 AI
MPO	< 0.2	< 0.4 U negative
Anti-PR3	< 0.2	< 0.04 U -ve
B2GP1, IgG	< 9	0–20 IgG Units
B2GP1, IgM	< 9	0–32 GPI IgM Units
Sm Ab	< 0.2	0.0–0.9 AI
RNP Ab	< 0.2	0.0–0.9 AI
Cardiolipin Ab IgM	< 9	0–12 MPL U/mL
Cardiolipin Ab IgG	20	> 20–80 GPL U/mL

*Paraneoplastic autoantibody evaluation*		
NMO/AQP4 IgG CSF	Negative	N/A
NMO/AQP4 FACS	Negative	N/A
IL2	< 31.2	0.0–31.2 pg/mL
ANNA 1 (Hu)	Negative	N/A
ANNA 2 (RI)	Negative	N/A
ANNA 3 serum	Negative	N/A
PCA 1 (Yo) serum	Negative	N/A
PCA 2, serum	Negative	N/A
PCA T6, serum	Negative	N/A
Amphiphysin Ab serum	Negative	N/A
GRMP IgG	Negative	N/A
P/Q type Ca channel Ab	0.00	< 0.02 nmol/L
ACH1C ganglionic neuronal Ab	0.00	
NMDA R Ab	Negative	N/A
LGI1 IgG CBA	Negative	N/A
CASPR2 IgG	Negative	N/A
GABA B R Ab	Negative	N/A
AMPA R ab	Negative	N/A
GAD 65 Ab	Negative	N/A
MOG Ab	Negative	N/A
DPPX Ab IFA	Negative	N/A
GFAP IFA	Negative	N/A
mGluR1 Ab IFA	Negative	N/A
MOG ABW/REFL titer CSF	Negative	N/A

*Infectious evaluation*		
CMV IgM Ab	< 30	0.09–29.9 AU/mL
CMV quant	Negative	N/A
EBV IgM ab	< 36	
EBV IgG Ab	> 600	0.0–17.9 U/mL
EBV nuclear Ag Ab	422	0.0–17.9 U/mL
HTLV1/HTLV2 Ab	Positive	N/A
HTLV1 western blot	Positive	N/A
HTLV2 western blot	Negative	N/A
Infectious mononucleosis	Negative	N/A
Mumps IgG	Positive	N/A
PVDL9 IgG	0.2	0.0–0.8
PVDL9 IgM	0.1	0.0–0.8
Rubella IgG Ab	Positive	N/A
Rubeola IgG Ab	Positive	N/A
*Aureobasidium pullulans* Ab	Negative	N/A
*Aspergillus fumigatus* F2 Ab and all other subtypes	Negative	N/A
*Coccidioides* Ab	Negative	N/A
Cryptococcal Ag CSF	Negative	N/A
Histoplasma Ag urine	Negative	N/A
TB blood (TSPOT)	Negative	N/A
Syphilis	Negative	N/A
VDRL CSF	Nonreactive	N/A
TP-PA	Negative	N/A
*Anaplasma* PCR	Negative	N/A
*Babesia* PCR	Negative	N/A
*Ehrlichia* PCR	Negative	N/A
Adenovirus PCR	Negative	N/A
B-D glucan	< 31	< 80 pg/mL
Malaria blood smear	Negative	N/A
*Toxoplasma* CSF	Negative	N/A

## Data Availability

All relevant clinical details are included within the article. Additional de-identified information may be available from the corresponding author on request, subject to privacy and ethical considerations.
